# Soil temperature forecasting using a hybrid artificial neural network in Florida subtropical grazinglands agro-ecosystems

**DOI:** 10.1038/s41598-023-48025-4

**Published:** 2024-01-17

**Authors:** Seyed Mostafa Biazar, Hisham A. Shehadeh, Mohammad Ali Ghorbani, Golmar Golmohammadi, Amartya Saha

**Affiliations:** 1 Department of Soil, Water and Ecosystem Sciences, University of Florida, IFAS/RCREC, Ona, FL USA; 2 Department of Artificial Intelligence and Computer Science, College of Computer Science and Informatics, Amman Arab University, Amman, Jordan; 3 Department of Water Engineering, University of Tabriz, Tabriz, Iran; 4 Archbold Biological Station, Buck Island Ranch, Lake Placid, FL 33852 USA

**Keywords:** Climate sciences, Hydrology, Solid Earth sciences

## Abstract

Soil temperature is a key meteorological parameter that plays an important role in determining rates of physical, chemical and biological reactions in the soil. Ground temperature can vary substantially under different land cover types and climatic conditions. Proper prediction of soil temperature is thus essential for the accurate simulation of land surface processes. In this study, two intelligent neural models—artificial neural networks (ANNs) and Sperm Swarm Optimization (SSO) were used for estimating of soil temperatures at four depths (5, 10, 20, 50 cm) using seven-year meteorological data acquired from Archbold Biological Station in South Florida. The results of this study in subtropical grazinglands of Florida showed that the integrated artificial neural network and SSO models (MLP-SSO) were more accurate tools than the original structure of artificial neural network methods for soil temperature forecasting. In conclusion, this study recommends the hybrid MLP-SSO model as a suitable tool for soil temperature prediction at different soil depths.

## Introduction

Soil temperature (ST) is a critical determinant that strongly impacts many physical, chemical, and biological processes in soil. Many factors influence soil temperature, such as meteorology, topography, soil water content, soil texture and vegetation cover/type^1^). Ground soil temperature can differ substantially under various current weather conditions and weather regions and land cover types. Soil temperature plays a very important role in plant growth, crop yield and agricultural processes and as such can be a more important factor than surface air temperature in agricultural production^2–5^. Therefore, forecasting soil temperature could be of importance for water resources decision makers as it has implications for irrigation requirements and scheduling.

A wide variety of techniques are used to simulate soil temperature. Numerous recent investigations have delved into short to medium-length ST forecasts, focusing on two distinct approaches^6,7^. The initial type emphasizes employing statistical methods, such as numerical weather forecasting techniques, which presume that future ST data series will exhibit statistical alterations akin to past occurrences^8,9^. Models for extended forecasts often require substantial data, which typically might not be available^10–12^. Conversely, the second type involves utilizing artificial intelligence (AI) models^13–15^, Moreover, several research initiatives have characterized ST as a nonlinear physical phenomenon^16–19^. In recent decades, many studies have focused on soil temperature modelling and forecasting^20–24^. For instance, the spatial and temporal patterns of soil temperature were predicted based on topography, surface cover and air temperature using the empirical relationship between air and soil temperature^25^. Reference^26^ modelled soil temperature for a range of forest species composition, ages and management systems across southern Australia, and sensitivity analysis indicated that one of the most important inputs was air temperature. The support vector machine (SVM) approach has been applied to predict diverse parameters including but not limited to soil moisture prediction, forecasting of river water quality, pan evaporation, stream flow prediction, global solar radiation, daily dew point temperature estimation, and interior environment variables in greenhouses^27–31^.

Other studies have determined that artificial neural network (ANN) models produce more accurate results compared to multivariate linear regression models in forecasting daily soil temperature^17,32^. Monthly soil temperature was predicted based on various atmospheric variables using linear and nonlinear regression models and an artificial neural network; it was found that neural networks were more precise methods compared to linear and nonlinear regressions to predict soil temperature^19,33–35^. Another study showed that the developed ANNs were a useful modelling approach for the spatiotemporal prediction of monthly soil temperature^18^. The application of the multilayer perceptron (MLP) and the adaptive neuro-fuzzy inference system (ANFIS) was examined to predict daily soil temperature in Illinois, and it was concluded that the MLP showed more accurate results than the ANFIS^36^.

In a study by Ref.^1^, soil temperature at multiple depths was predicted using a hybrid artificial neural network model and firefly optimizer algorithm and it was found that the hybrid MLP-FFA hybrid model produced more accurate results compared to the MLP model. Reference^37^ proposed a hybrid optimization method, namely Hybrid Genetic Algorithm and Sperm Swarm Optimization (HGASSO). The idea of this method was to amalgamate the Genetic Algorithm (GA) operations, such as mutation, selection and crossover operations with the local search of Sperm Swarm Optimization (SSO). This method is tested on solving different well-known multi-model benchmark functions. The results of the HGASSO prove its accuracy over both standard SSO and GA, which outperformed them in the terms of quality of results and speed of convergence. In a different study, Ref.^38^ proposed a hybrid method, namely “PSO-BP” that combines PSO variant with “back-Propagation (BP)”. The terms of solution quality and convergence speed were tested on various classical models. Depending on the experimental results, the researchers had presented that the hybrid variant is better than both BP and PSO in the aforementioned metrics. Reference^39^ discussed a hybrid approach, namely “HPSO-DE” that combines PSO with DE. The proposed HPSO-DE was evaluated on various test bed models in the field of optimization. The experimental results proved that “HPSO-DE” was more accurate in generating a better set of solutions.

The objective of the present research is to develop artificial intelligence models which would predict soil temperatures at different depths of the soil using meteorological data in the subtropical ranchlands of South Florida.

The study carries out a comprehensive comparative analysis between the proposed machine learning models (the Classical Multi-layer perceptron and the integrated multi-layer perceptron with Sperm Swarm Optimization algorithm). In addition, different combinations of climate variables have been examined as inputs for the model including but not limited to air temperature, solar radiation, wind speed and relative humidity. For the purpose of this study, a hybrid artificial neural network model was coupled with Sperm Swarm Optimization (SSO) for modeling daily soil temperature at a depth of 5, 10, 20, and 50 cm. The results of this study suggested that the combination of the SSO and hybrid artificial neural network models was a more accurate tool than the original structure of these artificial neural network methods for soil temperature forecasting purposes. To the authors’ knowledge, this is the first attempt to utilize an integrated artificial neural network model with Sperm Swarm Optimization (SSO) as a predictor for soil temperature.

## Material and methods

### Data acquisition

**Figure 1:**
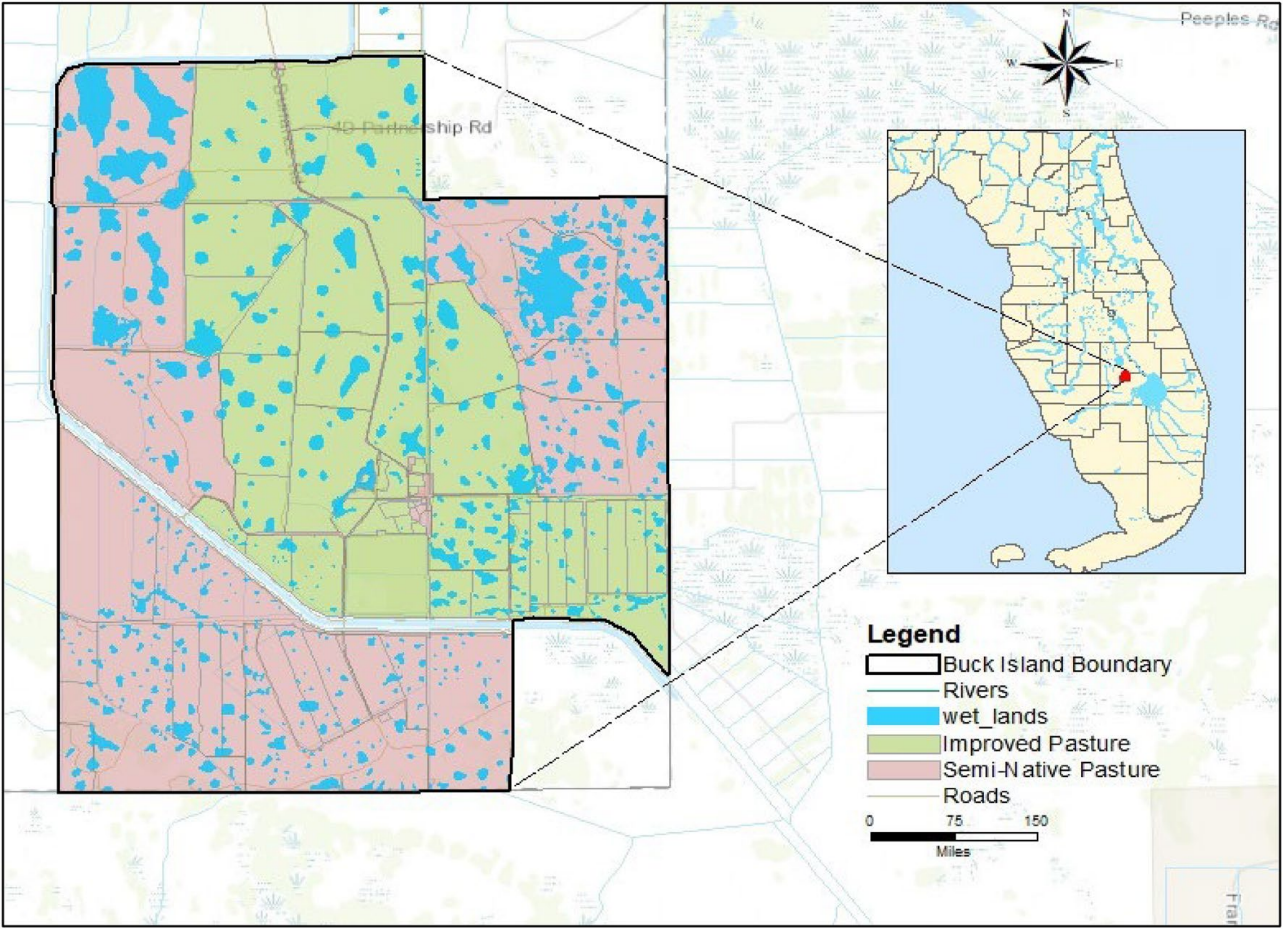
Study area location map.

The 4200 ha Buck Island Ranch (BIR), a division of Archbold Biological Station lies in Highlands County, Florida about 22 km southeast of Lake Placid within the headwaters of the Everglades in southcentral Florida (27° 09ʹ N, 81° 12ʹ W) (Fig. 1). It is a commercial free range cow-calf operation with improved (drained, fertilized, planted exotic grasses) and semi-native grasslands, seasonal wetlands and oak-palm forests. The climate is subtropical with average rainfall of 1360 mm and minimum and maximum temperatures of 15.9 and 29.0 °C (average of 30 years). Evapotranspiration is typically almost as high as rainfall^40,41^. The BIR Soils are sandy with an organic layer horizon on top. The Soils were dominated by Alfisols and Spodosols. In this region the seasonally inundated wetland–savanna mosaic has been drained by an extensive-ditch canal network constructed in the mid-twentieth century^42^.

The weather station at BIR measures rainfall (Texas Electronics TE25 tipping bucket Raingage), solar radiation (Kipp and Zonen pyranometers and pyrgeometers for short and longwave radiation), air temperature, relative humidity (Rotronic Hygroclip2 Temperature/RH Probe) and windspeed/direction (RM Young wind monitor). Soil temperature and moisture probes (Stevens Hydraprobe II) are placed at 5, 10, 20 and 50 cm depths. Data is recorded at 15-min intervals and stored in a datalogger (CR1000, Campbell Scientific).

For over three decades, Archbold’s Agroecology Program has been established at Buck Island Ranch. At this location, researchers collaborate with ranchers to comprehend the environmental effects of free-range cow-calf ranching and enhance its ecological sustainability. In 2012, Buck Island Ranch was chosen as one of 18 locations for the US Department of Agriculture’s Long-Term Agroecosystem Research (LTAR) network. LTAR employs synchronized research across different sites and shares data with the aim of enhancing the food system of America and increasing agricultural productivity. This initiative also seeks to ameliorate environmental quality amidst challenges such as climate change. Agroecological research at BIR is also relevant to subtropical grasslands and wetlands globally, with a lot of visitors and collaborations.

**Figure 2:**
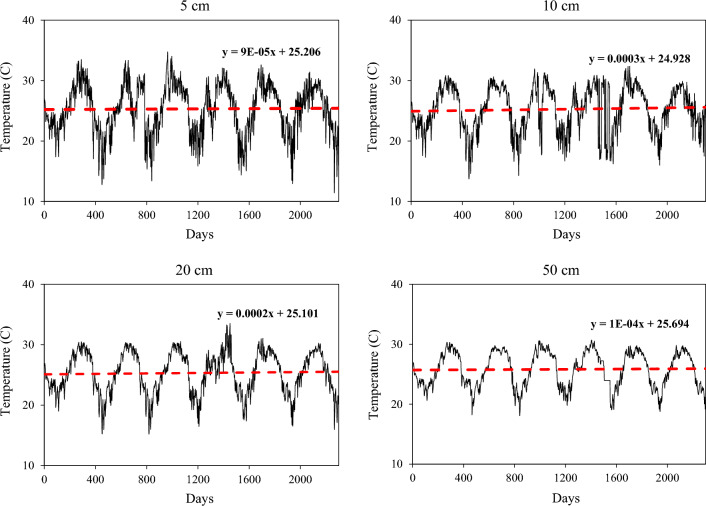
The soil temperature trends at different depths below ground surface.

Data collection and monitoring activities at Buck Island Ranch (BIR) were conducted over an extended period spanning from the 10th of December 2016 to the 27th of January 2023 (Table 1). This extensive time frame allowed us to gather a comprehensive set of data, reflecting both short-term variations and longer-term trends (Fig. 2).

**Table 1:** Basic statistics of meteorological variables for the period of 12/10/2016–27/01/2023.

Variables	Mean	Max	Min	Skewness
Air temperature (°C)	22.44	28.97	4.27	− 0.99
Wind speed (M/S)	2.2	10.33	0.38	1.83
Relative humidity (%)	80.27	99.21	47.82	− 0.45
Air pressure (mbar)	1016.58	1031.52	993.25	− 0.04
Rainfall (mm)	0.04	20.32	0.00	6.23
Solar radiation (W/m^2^)	443.76	491.51	341.50	− 0.78
Soil temperature (5 cm-°C)	25.31	34.75	11.41	− 0.43
Soil temperature (10 cm-°C)	25.25	32.37	13.73	− 0.42
Soil temperature (20 cm-°C)	25.32	33.54	15.19	− 0.36
Soil temperature (50 cm-°C)	25.81	30.71	18.1	− 0.29

### Methodology

#### Multi-layer perceptron neural networks (MLP)

The Multi-Layer perceptron (MLP), a form of the ANN model, will be adopted as the primary modeling tool to forecast soil temperature at multiple depths using a limited predictor dataset. In general, MLPs are extensively utilized for approximation, prediction, recognition and pattern classification. ANNMLP models can handle complex problems that are not linearly separable. Basically, the MLP model is a feed forward neural network with one or more layers among input and output layers^43^. The term feed forward signifies that the data feature extraction process moves in one direction from the input to output layer. The back propagation learning algorithm is used to train MLP^44–48^. Multilayer feed-forward Perceptron back propagation learning algorithm (MLP-BP), as one of the popular MLP architectures, involves input, hidden and output layers. Moreover, specific weights are linked among neurons of input and hidden layers and from neurons of hidden and output layers by suitable activation functions. Additionally, the activation functions between input and hidden layers and between hidden and output layers are sigmoid and linear functions, respectively. These activation functions limit the input data to fluctuate between 0 and 1. So, by assuming that input data = d = (Tmean, Patm, SR and M), the mathematical description is as follows:

1 $${d}_{j}={f}_{1}({b}_{j}+\sum_{i}^{I}{W}_{j,i}{d}_{i})$$

2 $${d}_{j}={f}_{2}({b}_{k}+\sum_{i}^{I}{W}_{kj}{d}_{j})$$

Where d is the array of input parameters including meteorological parameters; ${f}_{1}$ and ${f}_{2}$ are actuation functions, ${b}_{j}$ and ${b}_{k}$ are bias values of ${f}_{1}$ and ${f}_{2}$ and ${W}_{j,i}, {W}_{kj}$ are weight parameter.

In this study, backpropagation algorithm, which employs the extensively implements Levenberg–Marquardt, and the proposed SSO optimization algorithm were utilized for minimizing the error functions of MLP (Mean Squared Error (MSE) = $\frac{1}{n}\sum_{i=1}^{n}{({y}_{predicted}-{y}_{actual})}^{2}$, where n is the number of observation, ${y}_{predicted}$ predicted values and ${y}_{actual}$ observed values).

In this study we used 20 percent of data for the test period and 80% of data for training period same as^49,50^.

#### Standard “sperm swarm optimization (SSO)”

Sperm swarm optimization (SSO) is a newly developed metaheuristic algorithm that draws inspiration from the collective behavior exhibited by a group of sperm cells during the fertilization process of an ovum^51^.

The algorithm utilizes a collection of potential solutions, represented as "sperm," that traverse the entire search space in order to explore and acquire the optimal solution. Simultaneously, each candidate solution evaluates the best-performing sperm discovered thus far. In other words, a sperm takes into account it’s previously identified best position (sperm best solution) as well as the overall best position of the entire swarm (global best solution).

Within the SSO algorithm, each sperm enhances its position towards the optimal solution by taking into account its current location, velocity, the distance to its best solution (xbest_i_), and the distance to the global best solution obtained thus far (xgbest_i_). Mathematically, in SSO, the update of the sperm's position is governed by the following equation:

3 $${x}_{i}\left(t+1\right)={x}_{i}\left(t\right)+{v}_{i}(t)$$

Where ${x}_{i}\left(t\right)$ represents the current position of the ith sperm in the search space at time t, ${v}_{i}\left(t\right)$ denotes the velocity of the ith sperm at time t, which governs its movement, ${x}_{i}\left(t+1\right)$ is the updated position of the ith sperm at the subsequent time t + 1.

The equation provided represents the update of the current velocity ${v}_{i}(t)$ of the ith sperm in the algorithm. The velocity is comprised of three components: the initial velocity, the personal best solution (xbest_i_) of the sperm, and the global best solution (xgbest_i_), as depicted in Eq. (4).

4 $${v}_{i}(t) =\mathrm{ Initial}\_\mathrm{Velocity }+\mathrm{ Current}\_\mathrm{Best }+\mathrm{ Global}\_\mathrm{Best}$$

Where ${v}_{i}(t)$ is the velocity of the ith sperm at time tt, which directs its movement across the search space, $\mathrm{Initial}\_\mathrm{Velocity}$ denotes the inherent or starting velocity of the ith sperm, which can be thought of as the sperm's intrinsic momentum prior to any interactions or learning, $\mathrm{Current}\_\mathrm{Best}$ represents the influence of the best position that the ith sperm has discovered up to time t. This component pulls the sperm toward the most promising areas it has personally encountered, $\mathrm{Global}\_\mathrm{Best}$ reflects the influence of the best position found by any sperm in the swarm up to time t. This component guides the sperm towards the best solutions found by the entire collective.

The initial velocity of each sperm after being ejaculated into the search space (referred to as the cervix area) is represented in the first part of Eq. (4). This velocity is influenced by the pH value and can be mathematically expressed as follows:

5 $$Initial\_velocity=D.{log}_{10}.(pH\_{Rand}_{1}).{v}_{i}$$

The equation involves the damping factor D, which is a random number ranging from 0 to 1. Additionally, pH_Rand_1_ represents a random number within the range of 7–14, symbolizing the pH value of the visited location. The second term in Eq. (4) represents the best position achieved by the sperm thus far, influenced by both pH and temperature. This term can be expressed in the following manner:

6 $$Current\_Best={log}_{10}(pH\_{Rand}_{2}) .{log}_{10}(Temp\_{Rand}_{1}).{(xgbest}_{i}-{x}_{i}(t))$$

The equation continues with the term involving $pH\_{Rand}_{2}$, which is a randomly generated number between 7 and 14. Additionally, Temp_Rand1 represents another random number within the range of 35.1–38.5, signifying the temperature value of the visited location.

The final term in Eq. (4) represents the best position among all the sperm, which is the one that is closest to the target. This position is determined and evaluated using the following expression:

7 $$Global\_Best={log}_{10}(pH\_{Rand}_{3}).{log}_{10}.(Temp\_{Rand}_{2}).{log}_{10}.({xgbest}_{i}-{x}_{i}\left(t\right))$$

In this equation, pH_Rand_3_ represents a randomly generated number ranging from 7 to 14, and Temp_Rand_2_ represents another random number within the range of 35.1–38.5. By substituting Eqs. (5)–(7) into Eq. (4), the velocity of the ith sperm in iteration t can be defined as follows:

8 $${v}_{i}= D.{log}_{10}.(pH\_{Rand}_{1}).{v}_{i}+{log}_{10}(pH\_{Rand}_{2}) .{log}_{10}(Temp\_{Rand}_{1}).{(xgbest}_{i}-{x}_{i}(t))+{log}_{10}(pH\_{Rand}_{3}).{log}_{10}.(Temp\_{Rand}_{2}).{log}_{10}.({xgbest}_{i}-{x}_{i}\left(t\right))$$

In Eq. (8) integrates various components that represent the sperm’s movement in the search space influenced by environmental factors, namely pH and temperature.$D.{log}_{10}.(pH\_{Rand}_{1}).{v}_{i}$, This component represents the inherent momentum or initial velocity of the ith sperm, influenced by the pH of its immediate environment. The term ${log}_{10}.(pH\_{Rand}_{1})$ transforms a randomly selected pH value (ranging between 7 and 14) to introduce variability from the cervix area’s pH, with D being a damping factor that captures the natural variations in movement. Essentially, this captures the initial impetus a sperm has due to its immediate pH surroundings and ${log}_{10}(pH\_{Rand}_{2}) .{log}_{10}(Temp\_{Rand}_{1}).{(xgbest}_{i}-{x}_{i}(t))$, this term reflects the influence of both pH and temperature on the sperm’s current optimal position. It represents how these environmental factors affect the ability of the sperm to reach better positions. Then, ${log}_{10}(pH\_{Rand}_{3}).{log}_{10}.(Temp\_{Rand}_{2}).{log}_{10}.({xgbest}_{i}-{x}_{i}\left(t\right))$, This term determines the influence of pH and temperature on the global best position among all sperms, representing the overall optimal environmental conditions for the group.

In the Eq. (5) $Initial\_velocity$, describes the inherent momentum of a sperm immediately after its introduction into the cervix area (or the search space). Here, the pH value serves as an environmental factor influencing this initial movement. Specifically, the term ${log}_{10}.\left(p{H}_{{Rand}_{1}}\right)$ represents the logarithmic transformation of a random pH value between 7 and 14, modeling the variability of pH within the cervix area. Hence, the initial velocity is a function of both the randomly chosen pH value and the damping factor D, which represents natural variations. In Eq. (6) $Current\_Best$ signifies the optimal position a sperm has achieved, influenced by the interaction of pH and temperature. The multiplicative terms ${log}_{10}(pH\_{Rand}_{2})$ and ${log}_{10}(Temp\_{Rand}_{1})$ introduce variability from the randomly selected pH (between 7 and 14) and temperature values (between 35.1 and 38.5 °C), respectively. This equation captures the fact that a sperm’s performance (or ability to find better positions) is affected by both the pH and temperature of its current location. At the end in Eq. (7) $Global\_Best$ determines the superior position among all sperms, or the one nearest to the desired solution. Again, both pH and temperature values play vital roles. With terms like ${log}_{10}(pH\_{Rand}_{3})$ and ${log}_{10}.(Temp\_{Rand}_{2})$, we integrate the random effects of both pH and temperature on the collective performance of the sperm group^52–54^.

Within the SSO algorithm, as described in Eq. (8), the velocity of the sperm is influenced by two factors: the pH value and temperature of the visited zone. Temperature plays a crucial role as it allows the sperm to have awareness of the best solution, which corresponds to the location of the egg^55^.

#### Forecasting development

In this study, daily meteorological variables were obtained from 15-min measurements at the BIR weather station from 10th December 2016 to 27th January 2023. The selected meteorological variables are Air Temperature (°C) (T_mean_), Wind Speed (m/s) (Ws), Relative Humidity (RH), Air Pressure (mb), Rainfall (mm) (R), Solar Radiation (W/m^2^) (S_r_), Soil temperature (°C) (ST_5_) at 5 Soil temperature (°C) (ST_10_) at 10 cm Soil temperature (°C) (ST_20_) at 20 cm depth, and Soil temperature (°C) (ST_50_) at 50 cm depth. The statistical properties are displayed in Table 1. It is interesting to note that, the authors reviewed various papers and selected the most impactful variables from the literature, emphasizing commonly available ones^7,56,57^. Soil temperature mirrors air temperature at the surface, but deeper layers are more stable and lag behind in seasonal shifts observed at the top^1^. Wind speed impacts surface soil temperature through evaporation and moisture content. Its effect diminishes with depth and varies seasonally, influenced by factors like vegetation and solar radiation^58^. Higher relative humidity retains soil moisture, cooling surface soil. Low humidity can warm surface soil faster. Deep layers remain largely unaffected^35^. High air pressure can boost surface soil temperatures via clear skies and increased sun exposure, while deeper soil layers remain mostly unaffected^1^. Rain cools the topsoil directly, while deeper soil layers show little immediate temperature shifts from precipitation^26^. Solar radiation heats the soil surface directly. As depth increases, the influence of solar radiation on soil temperature diminishes^59^.

To increase model accuracy and to avoid selecting irrelevant input variables, Gamma Test (GT) was applied as a popular input variables selection (IVS) method.

The GT (Gamma Test) is employed to analyze the connection between inputs and outputs within numerical datasets. This approach differs significantly from previous non-linear analysis methods. In this method, a data sample is represented by a certain format^60,61^.

9 $$(\left({x}_{1},\dots ,{x}_{T}\right),y)$$

In this context, the input vector X is restricted to a closed bounded set C ϵ ${R}^{T}$, while the output is represented by the scalar y. For simplicity, the explanation focuses on the case of a single scalar output y. However, it is important to note that the same algorithm can be applied to scenarios where y is a vector without significant additional complexity or time overhead. The purpose of the GT is to provide an estimation of the noise variation, represented as Var(r), based on the data. The main assumption in this approach is that the system's underlying relationship follows a specific form.

10 $$y=f\left({x}_{1},\dots ,{x}_{T}\right)+r$$

In the given context, the variable r signifies an indeterminable component that can arise from either real noise or an insufficient functional determination within the input/output relationship. This component represents the unexplained or uncertain aspect of the system. Despite the unknown nature of the underlying function f, the GT is capable of directly estimating Var(r) using the available data. This estimation, referred to as the Gamma statistic (symbolized by γ), can be computed directly from the data with a time complexity of O (T log T). To compute γ, two specific quantities are derived through the following calculations:

11 $${\delta }_{t}(k)=\frac{1}{T}\sum_{i=1}^{T}\left|{x}_{N}\left[i,k\right]-{x}_{i}\right|$$

In the provided context, ${x}_{N}\left[i,k\right]$ refers to the index of the k-th nearest neighbor to xi, and |. | represents the Euclidean distance. The GT relies on the values of N [i, k], which represent the indices of the kth nearest neighbors (${x}_{N}\left[i,k\right]$) for each vector ${x}_{i}$ (with i ranging from 1 to T) typically with a value of p equal to 10. As a result, ${\delta }_{t}(k)$ represents the mean square distance to the kth nearest neighbor. The corresponding Gamma function of the output values is then determined.

12 $${\gamma }_{T}\left(k\right)=\frac{1}{2T}\sum_{i=1}^{T}{(yN\left[I,K\right]-{y}_{i})}^{2}$$

Using the GT, the mean-squared distances of the kth nearest neighbors (${\delta }_{t}(k)$) and the corresponding $\gamma {(p)}^{2}$ values up to a maximum value kMax are calculated. Subsequently, the regression line is computed, and the vertical intercept ($\Gamma$) is obtained as the Gamma value. Additionally, the slope A of the regression line is provided as an indication of the model's complexity (f). In theory, Γ represents the limit of $\gamma$ as the distances ($\delta$) approach zero, which corresponds to Var(r).

The GT is utilized as a preliminary step before modeling to estimate the variance of the output that cannot be explained by any smooth model based solely on the inputs, despite the unknown nature of the model itself. The GT helps capture the unexplained variability in the output. The estimation of error variance establishes a goal for the mean squared error that any smooth non-linear model should reach when applied to unseen data^33,50^.

Based on Table 2, the bolded variables were applied to predict ST in the cited depths (5, 10, 20 and 50 cm).

**Table 2:** GT values of all variables with soil temperature values at different depths (5–50 cm).

No	Variable	ST-5 cm	ST-10 cm	ST-20 cm	ST-50 cm
1	–	0.0399	0.0636	0.0420	0.0425
2	Air temperature	**0.0507**	**0.0742**	**0.0540**	**0.0507**
3	Wind speed	**0.0432**	**0.0643**	0.0419	0.0410
4	Relative humidity	**0.0465**	**0.0775**	**0.0531**	**0.0449**
5	Air pressure	**0.0466**	**0.0695**	**0.0457**	0.0414
6	Rainfall	**0.0449**	**0.0642**	**0.0423**	**0.0431**
7	Solar radiation	**0.0435**	**0.0766**	**0.0499**	**0.0483**

Significant values are in bold.

For ST prediction (in 5 and 10 cm) the whole meteorological variables were selected by GT (T_mean_, Ws, RH, P, R and S_r_). For two other depths, the ST values were estimated based on RH, R, and S_r_. It is interesting to note that GT recognized Air pressure (P) variable, as input variable, in addition to what was mentioned before to predict ST_20_. In this study, before training, the data was normalized by the approach proposed by^1^. Moreover 80% and 20% of data were used for training and testing, respectively same as suggested by^5,15^.

#### Performance evaluation

Understanding and predicting soil temperature is paramount due to its significant impact on various environmental, agricultural, and hydrological processes. Accurate models are thus vital for several practical applications, from agricultural decision-making to climate studies. Assessing model performance specifically for soil temperature prediction ensures that these models are both reliable and robust, providing stakeholders with trustworthy information for their respective uses. Several statistical indices including mean absolute percentage error (MAPE), root mean square error (RMSE), mean absolute error (MAE) and mean bias error (MBE) were used to evaluate the performance of the models (MLP and MLP-SSO), Correlation Coefficient (CC). These metrics collectively offer a comprehensive assessment of model performance, capturing both magnitude and direction of prediction errors, as well as potential biases. Their combination ensures a holistic evaluation, making certain that the model is reliable across various dimensions of accuracy^1,15^.

Furthermore, MAPE, Provides a relative measure of prediction accuracy, essential for understanding deviations in percentage terms, and its implication on a broader scale. RMSE, Ensures our model's precision, highlighting even occasional large errors which could be crucial for applications demanding high accuracy. MAE, Offers an average of the model's accuracy, ensuring its consistency across predictions. MBE, Monitors potential systematic biases, preventing consistent overpredictions or underpredictions which can skew decision-making. CC, Assesses the linear relationship between predicted and observed soil temperatures, ensuring the model effectively tracks variations^48,49^.

9 $$\mathrm{MAPE}=\frac{1}{n}\sum_{i=1}^{n}\left|\frac{{P}_{i}-{O}_{i}}{{O}_{i}}\right|\times 100$$

Where ${P}_{i}$ represents predicted values, while ${O}_{i}$ represents observed values.

The MAPE is the most common metric used to forecast error since the variable’s units are scaled to percentage units. The lower the value for MAPE the better, MAPE provides an understanding of prediction accuracy as a percentage. It measures the average absolute percent difference between observed and predicted values relative to the observed values. A reduction in MAPE indicates a higher prediction accuracy. For instance, a MAPE of 5% means that, on average, the model's predictions deviate from the actual observations by 5%. A decrease in this value means the model is becoming more precise in its predictions in percentage terms, which can be especially useful for relative comparisons and understanding the scale of prediction errors in proportion to actual values^62,63^

10 $$\mathrm{RMSE}=\sqrt{\frac{1}{N}\sum_{i=1}^{N}{({P}_{i}-{O}_{i})}^{2}}$$

Where ${P}_{i}$ represents predicted values, while ${O}_{i}$ represents observed values.

RMSE is frequently used measures of the differences between observed and predicted values. The unit of RMSE is the same as observed/predicted unit. The lower the value for RMSE the better Measures the square root of the average squared differences between predicted and observed values. RMSE gives more weight to larger errors than MAE, making it sensitive to occasional large errors. A reduction in RMSE suggests that the model is making fewer large errors, which is especially crucial when outliers or extreme values can have significant implications^9,64^.

11 $$\mathrm{MAE}=\frac{1}{N}\sum_{i=1}^{N}\left|({P}_{i}-{O}_{i})\right|$$

where ${P}_{i}$ represents predicted values, while ${O}_{i}$ represents observed values.

MAE in statistics is a measurement used to investigate how predictions are close to eventual outcomes. The unit of MAE is the same unit as the data being measured, Represents the average absolute differences between the observed and predicted values. A reduction in MAE indicates that the model's predictions are, on average, closer to the actual observations. For instance, a decrease in MAE by 2% means that the model's predictions are now, on average, 2% closer to the true soil temperature values, which can have tangible benefits in applications where precision matters^65^.

12 $$\mathrm{MBE}=\frac{\sum_{i=1}^{n}({O}_{i}-{P}_{i})}{N}$$

Where ${P}_{i}$ represents predicted values, while ${O}_{i}$ represents observed values.

MBE captures the average bias in the prediction. MBE is essentially applied to estimate the average bias in the model and to decide if any stages needed to be taken to modify the model bias. Indicates the average bias in the model predictions. A positive MBE suggests the model tends to overpredict, while a negative MBE indicates underprediction. Reducing the absolute value of MBE ensures that the model is not systematically biased in its predictions^65,66^.

The correlation coefficient (CC) quantifies the strength and direction of a relationship between two variables, A measure of the linear relationship between the observed and predicted soil temperatures. A coefficient value closer to 1 indicates a strong positive linear relationship, meaning that as observed temperatures increase, the model's predictions also tend to increase in a consistent manner. A high correlation suggests the model can effectively track changes in soil temperature^12^.

13 $$\mathrm{CC}=\frac{\sum ({x}_{i}-\overline{x })({y}_{i}-\overline{y })}{\sum {({x}_{i}-\overline{x })}^{2}\sum {({y}_{i}-\overline{y })}^{2}}$$

Where ${y}_{i}$ is the predicted values and ${x}_{i}$ is the observed values. $\overline{x }$ is the mean of observed values and $\overline{y }$ is the mean of predicted values.

In addition to statistical indices (Eqs. 9–13), a graphical method of Taylor diagram is used to illustrate the degree of correspondence between the observed and predicted behavior in terms of three statistics: the Pearson correlation coefficient (In the Taylor diagram, the angular position [azimuthal angle (angle from x-axis shows correlation. Increasing angle means decreasing correlation; on x-axis, it's perfect.)] represents the correlation coefficient. The Taylor diagram is instrumental in assessing model performance as it concisely visualizes key statistical measures—correlation, standard deviation, and RMSE—in one graphic. On the diagram, the azimuthal angle depicts correlation, the radial distance from the origin shows the normalized standard deviation, and the distance from a model point to a reference point indicates RMSE. This holistic representation provides quick insights into both the magnitude and pattern of model errors, aiding in the comparative evaluation of models or model configurations against reference data^67^. Models that reproduce the spatial pattern of the reference data will lie closer to the horizontal rightmost axis, indicating higher correlation), the root-mean-square error (RMSE) (In the diagram, the distance from a model point to the reference point (usually set at (1,0) for normalized plots) represents the RMSE. Points closer to the reference point have smaller RMSE values, denoting better agreement with observations), and the normalized standard deviation [the radial distance (distance from center shows model's deviation. Perfect match is on radius 1. Inside: underestimation, outside: overestimation) from the origin in the Taylor diagram represents the normalized standard deviation. A value equal to the reference standard deviation implies that the model has accurately captured the observed variability, while values above or below indicate overestimation or underestimation, respectively] in a single diagram. The Taylor diagram is a graphical tool developed by Karl Taylor in the late twentieth century, designed to provide a comprehensive visual summary of how closely a model’s pattern matches observations. Instead of multiple plots to compare various metrics, the Taylor diagram condenses this information into a single plot, making the assessment of multiple models more straightforward. Taylor diagram can be displayed as a series of points on a polar plot. The azimuth angle implies the Pearson Correlation ® value between the estimated and observed data. The radial distance from the origin, meanwhile, signifies the ratio of the normalized standard deviation (SD) of the simulation to that of the observation. The centered RMSE in the simulated field is proportional to the distance from the point on the x-axis^58,67^.

## Results and discussion

This section delves into a comprehensive comparison between the hybrid MLP-SSO model and its classical counterpart, the MLP model, in predicting soil temperatures at varying depths. Through statistical indicators and graphical presentations, we aim to highlight their respective efficiencies.

The performance of both hybrid MLP-SSO model and classical MLP model are presented using the statistical indices and visual assessment of predicted and observed soil temperature data at different depths. Table 3 presents the comparison of the performances of the MLP and MLP-SSO for the model development (training) and model validation (Testing) datasets. Both models were evaluated at the depths of 5, 10, and 20, 50 cm depths with statistical criteria (RMSE, MAE, MAPE, and MBE).

**Table 3:** Performance criteria of the MLP-SSO and MLP models for training and testing stages at the Buck Island Ranch station.

Models		Depth	Structures	CC	RMSE (°C)	MAE (°C)	MAPE %	MBE (°C)
Train	MLP	5	(6-3-1)	0.897	1.843	1.263	2.904	0.018
		10	(6-4-1)	0.816	2.237	1.452	3.476	− 0.223
		20	(5-10-1)	0.895	1.561	1.177	2.793	− 0.034
		50	(4-2-1)	0.899	1.286	0.962	2.254	0.011
	MLP-SSO	5	(6-3-1)	0.897	1.839	1.261	2.904	− 0.019
		10	(6-4-1)	0.822	2.192	1.459	3.466	0.000
		20	(5-10-1)	0.883	1.641	1.240	2.940	− 0.011
		50	(4-2-1)	0.895	1.307	0.983	2.303	− 0.004
Test	MLP	5	(6-3-1)	0.940	1.347	0.993	2.419	− 0.114
		10	(6-4-1)	0.881	1.417	1.065	2.561	− 0.153
		20	(5-10-1)	0.953	1.01	0.791	1.921	0.042
		50	(4-2-1)	0.921	1.233	0.916	2.188	− 0.193
	MLP-SSO	5	(6-3-1)	0.941	1.332	0.993	2.364	− 0.084
		10	(6-4-1)	0.896	1.367	1.035	2.502	− 0.142
		20	(5-10-1)	0.955	0.973	0.758	1.840	− 0.028
		50	(4-2-1)	0.935	1.232	0.927	2.208	− 0.254

The statistical analysis results which are presented in Table 3 show that during the testing period, the RMSE values for different depths for MLP model are estimated in the range of 1.01–1.417 ($^\circ{\rm C}$), while the RMSE values for the MLP-SSO model was figured out to be between 0.973 and 1.367 ($^\circ{\rm C}$).

At the 5 cm depth, the MLP-SSO results outperformed the classical MLP model over the testing period. The MLP-SSO model assigned an RMSE of 1.332 (°C), MAE of 0.993 (°C), MAPE of 2.364% and MBE of − 0.084 (°C). In other words, the integration of SSO algorithm with MLP model led to reduction in the RMSE and MAE, 1.10% and 2.35% respectively. Based on this metrics, the new model improved the accuracy. Araghi et al. 2017 had same results.

At the depth of 10 cm the MLP-SSO model produced superior results in comparison with MLP. The results showed the RMSE of 1.367 (°C), MAE of 1.035 (°C), MAPE % of 2.502 and MBE of − 0.142 (°C) for MLP-SSO. Moreover, the MLP-SSO generated lower values of the RMSE and MAE rather than the classical MLP model. The MLP-SSO model reduced them by 10.1% and 2.9%, respectively, same as^68^ outputs.

**Figure 3:**
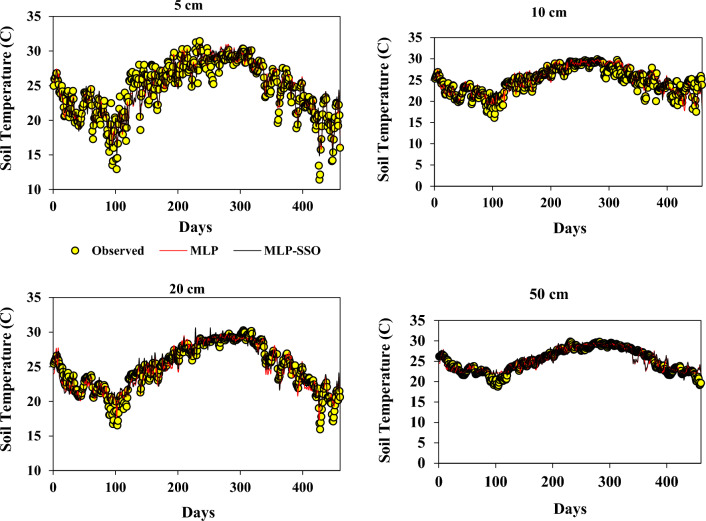
Observed and predicted ST values by MLP and MLP-SSO models in the test period.

Figure 3 displays a comparative time series of projected versus actual soil temperature (ST) values at varying depths, as determined by both the MLP and MLP-SSO models. This illustration provides a straightforward visual juxtaposition of predictions from the two models in relation to the true data over time.

The same trend can be seen for at the 20 cm depth. At this depth, the results of MLP-SSO is more precise than the classical MLP model. The MLP-SSO yielded an RMSE value of 0.973 (°C), MAE of 0.758 (°C), MAPE of 1.840% and MBE of -0.028 (°C). Based on aforementioned values, the RMSE and MAE were reduced by about 2.31% and 4.14%, respectively. Samadianfard et al. 2018, reached out to the same results.

However, dissimilar, the trend found for the other depth. The MLP-SSO model showed different results in modeling the ST at 50 cm depth. The MLP-SSO model reduced RMSE from 1.233 in the classical MLP model to 1.232 in the hybrid MLP-SSO model, but it can be seen an increasing trend in the other criteria.

Based on the statistical analysis conducted in this study, it can be concluded that the SSO-based model had a remarkable effect in reducing the predicting errors for ST at the 5, 10 and 20 cm depths below the soil surface. While the MLP-SSO model was unable to enhance the accuracy of ST at 50 cm depth. This conclusion concurs with^1^. Even^17^ results proved that when depth increased the model accuracy decreased.

**Figure 4:**
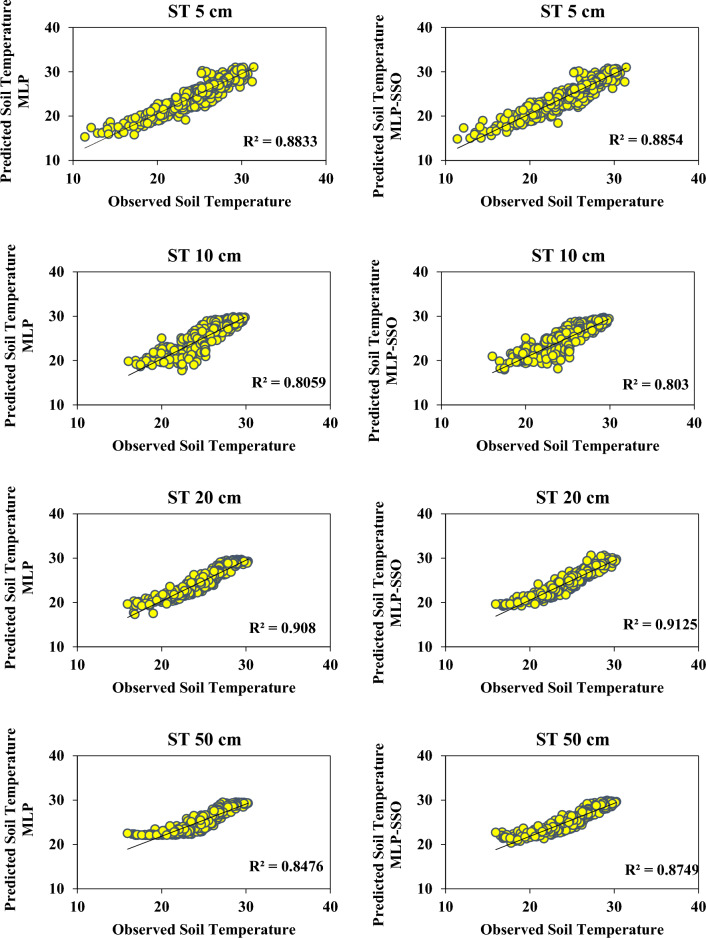
Scatterplots of the predicted-observed ST for test section.

**Figure 5:**
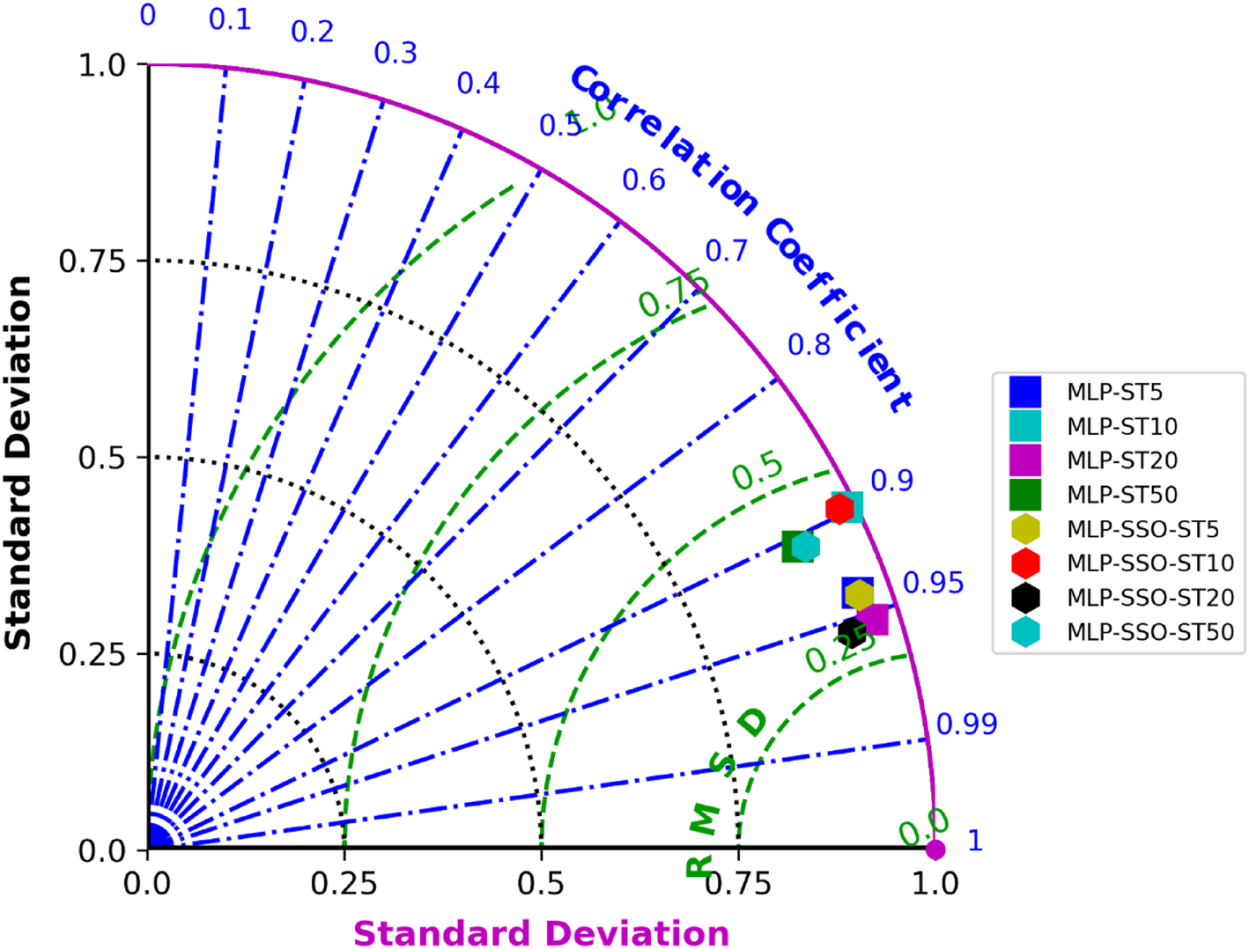
Taylor diagram of the predicted ST values in test period.

Figure 3 presents a time series comparison of predicted and observed soil temperature (ST) values at different depths using both MLP and MLP-SSO models. This Figure offers a direct visual comparison of the two model predictions over time against actual observations. Figure 4 provides scatterplots to visually contrast the predicted ST values from the models against observed ST values. The scatterplots highlight the accuracy and fit of each model, with the tighter clustering of points indicating a better model fit. This graphically showcases the superiority of MLP-SSO over the traditional MLP models, especially when assessing the performance across various soil depths. Figure 5 displays the Taylor diagram, which is instrumental in evaluating the performance of the two models at multiple depths. The diagram employs reference points to indicate centered RMSE differences, and the distance from these points signifies model accuracy. Models closer to the reference point with a correlation coefficient of 1, possessing a similar range of variations as the observations, are considered superior. In this diagram, it's evident that the MLP-SSO model, represented by circles, consistently outperforms the classical MLP model, denoted by squares, across all soil depths in terms of prediction accuracy.

It can be seen in Fig. 3 the time series of predicted and observed ST values at different depths with the MLP and MLP-SSO are demonstrated. In the Fig. 4 the Scatterplots of predicted and observed ST values are illustrated. The superiority of MLP-SSO over the MLP models is proved with these graphs. Comparing of the model predicted for different depths, as displayed in Fig. 4. Obviously illustrates that MLP-SSO model able to estimate the soil temperature values better than classical MLP models.

It can be seen in Fig. 5 the Taylor diagram for both models utilized at multiple depths. In this diagram, the distance from, reference point (i.e. a hollow point) is an amount of the centered RMSE difference. Accordingly, a premier model is normally demonstrated by the reference point with a correlation coefficient of 1 with nearly the same domain of variations compared with the observations. It is obvious from Fig. 5 that the MLP-SSO (i.e., Circle) was able to obtain high accuracy predicts of soil temperature rather than the classical MLP model (i.e., Square) applied at all soil depths.

The enhanced accuracy in soil temperature prediction achieved through our MLP-SSO model can substantially benefit agricultural practices, especially in precision farming where optimal planting and irrigation schedules are determined by soil temperature data. Such accurate predictions can also optimize water resource management and provide invaluable insights for climate change research, especially in modeling carbon and nitrogen cycling in ecosystems, thus refining greenhouse gas emission forecasts from soils. Nevertheless, while our model excelled in Florida's subtropical grazinglands, its performance might differ in areas with distinct climate or soil characteristics. Additionally, its effectiveness is tied to the quality and consistency of the input meteorological data. Thus, while promising, users should account for local conditions and ensure robust input data when leveraging the MLP-SSO model for practical applications.

In our exploration of predicting soil temperatures across various depths, the study underscored the pivotal role of soil temperature as a determining factor for numerous soil-based reactions. Evaluating the predictive accuracy of two intelligent neural models in the subtropical grazinglands of Florida, it was evident that the combined prowess of artificial neural networks (ANNs) and Sperm Swarm Optimization (SSO) resulted in the MLP-SSO model. This hybrid model notably surpassed the traditional artificial neural network methods in forecasting soil temperature, offering a significant improvement in predictive capability. Utilizing a comprehensive seven-year meteorological dataset from Archbold Biological Station, the performance metrics clearly showcased the MLP-SSO model's superior precision. In essence, for those looking to predict soil temperature across various depths, especially in regions with similar environmental dynamics to South Florida, the hybrid MLP-SSO model emerges as a highly recommended tool, outclassing the classical MLP models in accuracy and reliability.

Future research endeavors could delve deeper into refining and expanding the MLP-SSO model by integrating it with other optimization techniques or newer neural network architectures. This could further enhance its prediction accuracy for soil temperatures across diverse geographical landscapes and climatic conditions. Additionally, the influence of different land cover types on soil temperature prediction warrants comprehensive investigation. While the current model has been tested extensively with data from subtropical grazinglands of Florida, its adaptability and efficiency in other climatic zones remain an area worth exploring. Another promising avenue would be the inclusion of more environmental variables into the model, potentially offering a holistic understanding of their collective impact on soil temperature variations. Lastly, assessing the real-time applicability of the MLP-SSO model in agricultural, ecological, or urban planning scenarios could provide actionable insights for stakeholders and drive innovations in the field of soil temperature prediction.

## Conclusion

Accurate soil temperature predictions are pivotal for strategic decision-making in agriculture and water resource management. Our study introduced and validated a novel hybrid model, MLP-SSO, for forecasting soil temperatures at varied depths at Buck Island Ranch, South Florida. When compared with the conventional MLP model, the MLP-SSO demonstrated superior predictive accuracy and efficiency, especially when calibrated using readily accessible meteorological variables from 2016 to 2023.

The significant edge of the MLP-SSO underscores its potential as a premier tool in anticipating irrigation needs, especially with agriculture's burgeoning water demands. Beyond irrigation, the model holds promise for applications in understanding forest/grassland productivity dynamics and aiding fire management strategies. Future iterations of the MLP-SSO could explore incorporating additional climatic or soil health/type variables to enhance prediction finesse. This research holds tangible value for stakeholders, from water resource managers and farmers to environmental researchers, enabling them to harness data-driven insights for optimal resource management and sustainable agricultural practices. In the realm of policy, the precision of the MLP-SSO model could inform frameworks focused on sustainable water utilization and soil health management in agriculture. In essence, the MLP-SSO model emerges not just as an academic advancement but as a keystone for future-ready, sustainable agriculture.

In conclusion, the results of current study recommend that the hybrid MLP-SSO model could be a suitable tool for soil temperature prediction at different soil depths. Being calibrated with easily available weather data, this tool can be utilized to forecast and anticipate irrigation demand by water resource managers, given the large and increasing demand of water from agriculture amidst scenarios of decreasing water availability. Studies exploring the connection of soil temperature with forest/grassland productivity, fire management and land use change can also benefit from this tool. Soil temperature forecasting over wide areas with sparse meteorological stations can also inform evapotranspiration (ET) forecasts, given the direct link between soil temperature and ET. Given the magnitude of ET in subtropical and tropical watershed water balances, the relartion with land use change and the current uncertainty in estimating ET^69^, this tool can constrain this uncertainty to some extent, and thereby improve watershed water balance computations.

## Data Availability

The datasets used and/or analysed during the current study available from the corresponding author on reasonable request.
